# WTAP-Mediated m6A RNA Methylation Regulates the Differentiation of Bone Marrow Mesenchymal Stem Cells via the miR-29b-3p/HDAC4 Axis

**DOI:** 10.1093/stcltm/szad020

**Published:** 2023-04-03

**Authors:** Jincheng Liu, Yunhao You, Zhenqian Sun, Lu Zhang, Xiang Li, Zihan Dai, Jinlong Ma, Yunzhen Chen, Guangjun Jiao

**Affiliations:** Department of Orthopaedics, Qilu Hospital of Shandong University, Jinan, Shandong, People’s Republic of China; Department of Orthopaedics, Qilu Hospital of Shandong University, Jinan, Shandong, People’s Republic of China; Department of Orthopaedics, Qilu Hospital of Shandong University, Jinan, Shandong, People’s Republic of China; Department of Orthopaedics, Qilu Hospital of Shandong University, Jinan, Shandong, People’s Republic of China; Department of Orthopaedics, Qilu Hospital of Shandong University, Jinan, Shandong, People’s Republic of China; Department of Orthopaedics, Qilu Hospital of Shandong University, Jinan, Shandong, People’s Republic of China; Department of Orthopaedics, Qilu Hospital of Shandong University, Jinan, Shandong, People’s Republic of China; Department of Orthopaedics, Qilu Hospital of Shandong University, Jinan, Shandong, People’s Republic of China; Department of Orthopaedics, Qilu Hospital of Shandong University, Jinan, Shandong, People’s Republic of China

**Keywords:** WTAP, BMMSCs, m6A, osteogenesis, adipogenesis, miR-29b-3p, HDAC4

## Abstract

N6-methyladenosine (m6A) methylation, a well-known modification with new epigenetic functions, has been reported to participate in the progression of osteoporosis (OP), providing novel insights into the pathogenesis of OP. However, as the key component of m6A methylation, Wilms tumor 1-associated protein (WTAP) has not been studied in OP. Here we explored the biological role and underlying mechanism of WTAP in OP and the differentiation of bone marrow mesenchymal stem cells (BMMSCs). We demonstrated that WTAP was expressed at low levels in bone specimens from patients with OP and OVX mice. Functionally, WTAP promoted osteogenic differentiation and inhibited adipogenic differentiation of BMMSCs in vitro and in vivo. In addition, microRNA-29b-3p (miR-29b-3p) was identified as a downstream target of WTAP. M6A modifications regulated by WTAP led to increased miR-29b-3p expression. WTAP interacted with the microprocessor protein DGCR8 and accelerated the maturation of pri-miR-29b-3p in an m6A-dependent manner. Target prediction and dual-luciferase reporter assays identified the direct binding sites of miR-29b-3p with histone deacetylase 4 (HDAC4). WTAP-mediated m6A modification promoted osteogenic differentiation and inhibited adipogenic differentiation of BMMSCs through the miR-29b-3p/HDAC4 axis. Furthermore, WTAP-mediated m6A methylation negatively regulates osteoclast differentiation. Collectively, our study first identified a critical role of WTAP-mediated m6A methylation in BMMSC differentiation and highlighted WTAP as a potential therapeutic target for OP treatment.

## Graphical Abstract

**Graphical Abstract:**
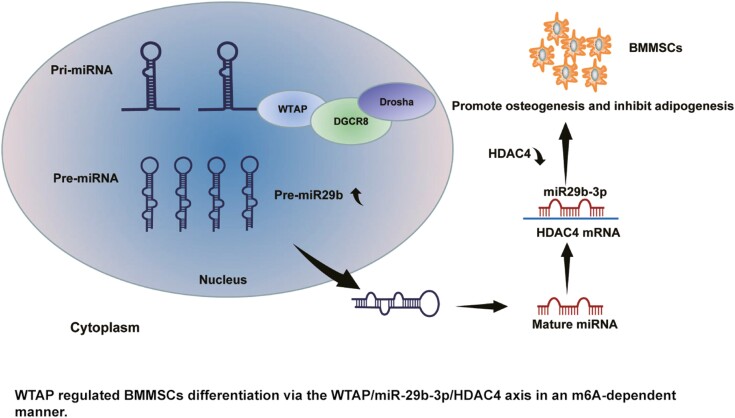

**Significance Statement**

With the aging of the general population, osteoporosis has become an increasingly serious public health problem. Bone loss caused by the differentiation of BMMSCs into adipocytes is an important mechanism of osteoporosis. A principal requirement for improving osteoporosis is the synthesis of metabolic agents capable of increasing bone formation and reducing fat accumulation by targeting BMMSCs. N6-methyladenosine (m6A) methylation is involved in regulating various biological processes in vivo, such as osteoporosis. In the present study, we found that WTAP-mediated m6A modification regulates the differentiation of BMMSCs through the miR-29b-3p/HDAC4 axis. WTAP may represent a promising target for osteoporosis treatment.

## Introduction

Osteoporosis (OP) is a common disease characterized by low bone mass, microarchitectural deterioration, and excessive adipose tissue accumulation in the bone marrow milieu, resulting in fragility fractures, disability, and death in elderly patients.^1-3^ OP can affect people of all ethnic backgrounds but is prevalent in postmenopausal women and older men.^1,4^ Although estrogen, calcitonin, and bisphosphonates are conventional and effective clinical treatments for OP, there is still no cure for this condition.^5^ Therefore, it is urgent to seek new therapeutic targets for OP.

The imbalance between osteogenic and adipogenic differentiation of bone marrow mesenchymal stem cells (BMMSCs) may affect the occurrence and development of OP.^6^ BMMSCs are the common progenitors of osteoblasts and marrow adipocytes.^7^ BMMSCs have multiple differentiation potentials and play an important role in maintaining normal bone stability.^8^ BMMSCs can reduce bone formation and accumulate marrow fat through a decreased capacity to differentiate into osteoblasts and increased capacity to differentiate into adipocytes, which subsequently promote OP development.^9^ However, the potential mechanism governing the balance between adipogenic and osteogenic differentiation of BMMSCs remains unclear.

N6-methyladenosine (m6A) RNA methylation is one of the most common modifications in eukaryotes, accounting for more than 60% of all RNA modifications.^10,11^ This modification is a reversible process that is modulated by m6A “reader,” “writer,” and “eraser” proteins.^12^ M6A was reported to be widely involved in OP development by regulating BMMSC differentiation.^13-15^ As one of the m6A methyltransferases, Wilms tumor 1-associated protein (WTAP) can regulate the recruitment of the m6A methyltransferase complex to mRNA targets, altering the progression of multiple diseases including carcinoma, degenerative disorders, and cardiovascular diseases.^16-19^ Recent research has shown that WTAP regulates the development of diseases by mediating glycolysis and lipid metabolism.^20-22^ Nevertheless, the role of WTAP in OP and the molecular mechanism by which WTAP-mediated methylation regulates BMMSC differentiation remain unclear.

Accumulating studies have shown that microRNAs (miRNAs) are responsible for regulatory functions at the post-transcriptional level by repressing target mRNA expression and play an important role in metabolism and BMMSC differentiation.^23-27^ Recent studies have also explored the relative mechanism by which methylation mediates BMMSCs differentiation by regulating the maturation of miRNAs.^14^ However, whether WTAP affects BMMSCs differentiation by regulating miRNA synthesis is still unclear.

In the present study, our studies demonstrated that WTAP promoted osteogenesis and inhibited adipogenesis of BMMSCs via the WTAP/miR-29b-3p/HDAC4 pathways in an m6A-dependent manner. Furthermore, WTAP could negatively regulate the osteoclast differentiation of macrophages. The results indicated that WTAP could be a novel therapeutic target for OP.

## Materials and Methods

### Human Bone Specimens

Ten bone specimens were obtained from female OP patients and from 10 female control subjects without OP and other bone-related anomalies. The specimen collection was conducted by the Department of Orthopaedics, Qilu Hospital of Shandong University. This study was approved by the Medical Ethics Committee of Qilu Hospital of Shandong University.

### Cell Culture

The murine primary BMMSCs were obtained from Zhong Qiao Xin Zhou Biotechnology Co., Ltd. (#ZQ0465, Shanghai, China). BMMSCs were cultured in DMEM/F12 (1:1) medium (#11320033, Gibco, US) containing 10% fetal bovine serum (FBS) and 1% double antibiotics (penicillin/streptomycin mix) (#10378016, Gibco, USA). The BMMSCs were maintained in a humidified atmosphere of 5% CO_2_ at 37 °C. RAW264.7 cells were purchased from Zhong Qiao Xin Zhou Biotechnology Co., Ltd. (#ZQ0098, Shanghai, China), cultured with DMEM supplemented with 10% FBS and 1% antibiotics, and incubated in a humidified atmosphere of 5% CO_2_ at 37 °C. For osteoclast differentiation induction, RAW264.7 cells were treated with 50 ng/mL RANKL (#CJ94, Novoprotein, Beijing, China).

### Mice Model of OP

Experiments involving the mice model of OP were performed in accordance with the Guide for the Care and Use of Laboratory Animals and approved by the Institutional Animal Use and Care Committee of Qilu Hospital of Shandong University. C57BL/6 mice (female, 3 months old, 5 mice per group) were purchased from the Animal Experiment Center of Shandong University (Jinan, China). After the mice were anesthetized with 3% sodium pentobarbital, a 20 mm incision was carefully cut on the lumbar back of the animal and the surrounding tissue was dissociated to expose the ovaries. Then, we removed the bilateral ovaries after clamping the blood vessels and fallopian tubes for mice in the ovariectomized (OVX) group. We removed the nearby adipose tissue of the bilateral ovaries from the mice in the sham-controlled group (Sham). Two months after the OVX model establishment, the OVX mice receive lentivirus treatment.

### RNA Extraction and Quantitative Real-Time PCR (qRT-PCR)

TRIzol (#15596026, Invitrogen, Carlsbad, CA, USA) was used to extract total RNA from BMMSCs and fresh bone tissues. According to the manufacturer’s instructions, the Biospin miRNA Extraction Kit (#BSC64S1, Bioer Technology, China) was used to extract miRNAs. A ReverTra Ace qPCR RT Kit (#TRT-101, Toyobo, Japan) and Mir-X miRNA First-Strand Synthesis kit (#638315, TaKaRa, USA) were used to synthesize complementary DNA from total RNA and miRNA, respectively. qRT-PCR was conducted in a LightCycler 480 System (Roche, USA) using LightCycler480 software 1.5.1.62 SP3 to determine the mRNA and miRNA expression levels of various genes. GAPDH and U6 were used as internal standard controls for normalization by using the comparative CT method. The sequences of all primers used for qRT-PCR amplification are listed in Supplementary Table S1.

### RNA m6A Quantification

Total RNA was extracted from cells and tissues using TRIzol reagent (#15596026, Invitrogen, Carlsbad, CA, USA). The EpiQuik m6A RNA Methylation Quantification kit (colorimetric) (#P-9005-48, EpiGentek, USA) was used to measure the relative m6A content following the manufacturer’s protocol. A microplate reader was used to measure the absorbance at 450 nm, and the percentage of m6A in total RNA was calculated using the following formula: m6A% = [(sample OD − NC OD)/S]/[(PC OD − NC OD)/P] × 100%, where NC (negative control) indicates RNA without m6A, PC (positive control) represents m6A oligomers that are normalized to 100% m6A, S represents the amount of input sample RNA (ng), P is the amount of input positive control (ng), and PC represents the positive control.

### Western Blot Analysis

Total proteins were extracted from BMMSCs and bone samples using RIPA lysis buffer with phenylmethylsulfonyl fluoride (PMSF) (#ST506, Beyotime, China). Then, the proteins were quantified using the bicinchoninic acid (BCA) method (#P0012S, Beyotime, China). Equal amounts of protein samples were separated on a polyacrylamide gel and transferred onto a nitrocellulose membrane. After blocking with 5% fat-free dry milk at RT for 1 h, the obtained membranes were incubated with an anti-WTAP antibody (#41934S, 1:1000, Cell Signaling Technology, MA, USA), anti-RUNX2 antibody (#12556, 1:1000, Cell Signaling Technology, MA, USA), anti-ALP antibody (#DF6225, 1:1000, Affinity Bioscience, Jiangsu, China), anti-OCN antibody (#bs-0470R, 1:1000, Bioss, Beijing, China), anti-COL1 antibody (#ab270946, 1:1000, Abcam, Cambridge, UK), anti-LPL antibody (#ab91606, 1:1000, Abcam, Cambridge, UK), anti-PPARγ antibody (#2443S, 1:1000, Cell Signaling Technology, MA, USA), anti-C/EBPα antibody (#8178S, 1:1000, Cell Signaling Technology, MA, USA), anti-C/EBPβ antibody (#43095S, 1:1000, Cell Signaling Technology, MA, USA), anti-HDAC4 antibody (#7628, 1:1000, Cell Signaling Technology, MA, USA), anti-MMP-9 antibody (#ab76003, 1:1000, Abcam, Cambridge, UK), anti-NFATc1 antibody (#ab183023, 1:1000, Abcam, Cambridge, UK), anti-CTSK antibody (#ab187647, 1:1000, Abcam, Cambridge, UK), anti-C-FOS antibody (#ab302667, 1:1000, Abcam, Cambridge, UK), and anti-GAPDH antibody (#bsm-33033M, 1:5000, Bioss, Beijing, China) at 4 °C overnight. The treated membranes were then incubated with a secondary antibody (#7074S, 1:3000, Cell Signaling Technology, MA, USA). Finally, the antibody-antigen complexes were visualized with an Amersham ImageQuant 800 system (USA).

### Immunohistochemistry (IHC)

All mice femurs were formalin-fixed and paraffin-embedded by a routine method. After antigen retrieval, each section was incubated with anti-WTAP antibodies (#41934S, 1:200, Cell Signaling Technology, MA, USA) and anti-HDAC4 antibodies (#7628, 1:200, Cell Signaling Technology, MA, USA) at 4 °C overnight. The obtained sections were then incubated with secondary antibody (#7074S, 1:1000, Cell Signaling Technology, MA, USA) for 1 h at room temperature. The stained tissues were visualized under a microscope (Aoren, China) and analyzed by ImageJ software (NIH, Bethesda, MD, USA).

### Osteogenic and Adipogenic Differentiation of BMMSCs

BMMSCs were seeded into 6-well plates. Osteogenic differentiation was induced by DMEM/F12 medium containing 10 nM dexamethasone, 0.25 mM ascorbic, 10 mM β-phosphoglycerol, 1% L-glutamine, and 10% FBS. Afterward, the osteogenic-differentiated cells were stained with alizarin red stain (ARS) and alkaline phosphatase (ALP). Adipogenic differentiation was induced by DMEM/F12 medium containing 0.5 mM 3-isobutyl-1-methylxanthine, 10 μg/mL insulin, 1 μM dexamethasone, 0.2 mM indomethacin, 10% FBS, and 1% double antibiotics for 48 h. On the third day, 10 μg/mL insulin was added to the complete medium. This process was repeated for 10 days, and the cultured cells were stained with Oil Red O.

### ARS and ALP Staining

After 7 or 14 days of osteogenic differentiation induction, BMMSCs were fixed with 4% formaldehyde for 40 minutes and rinsed with phosphate-buffered saline (PBS) 3 times. Then, the cultured cells were stained with ARS (#G1450, Solarbio, Beijing, China) for 30 minutes at room temperature. ALP staining was performed using a BCIP/NBT alkaline phosphatase color development kit (#C3206, Beyotime, Shanghai, China) according to the manufacturer’s protocol.

### ALP Activity Assay

Each group of BMMSCs was plated into 6-well plates and cultured in an osteogenic differentiation medium containing different treatments. The cells were then immobilized, and ALP activity was measured using an ALP assay kit (#P0321S, Beyotime, Shanghai, China). Then, the absorbance was analyzed by a spectrophotometer (Bio-Rad) at 405 nm.

### Oil Red O Staining and Extraction

After 10 days of adipogenic differentiation induction, the cells were immobilized with 4% paraformaldehyde for 15 minutes. Then, the cells were stained with Oil Red O (#G1262, Solarbio Co., Beijing, China) at 37 °C for 15 minutes according to the manufacturer’s protocols. The cells were washed with distilled water and subsequently imaged under a microscope (Aoren, China). Isopropanol was used to extract the dye of Oil Red O-positive cells, and the OD value was analyzed by spectrophotometer (Bio-Rad) at a wavelength of 510 nm.

### Identification of BMMSCs Surface Antigen by Flow Cytometry

P3 BMMSCs were digested with trypsin and resuspended in DMEM containing 10% FBS. The cells were washed with phosphate-buffered saline (PBS) twice. Subsequently, the cells were incubated with anti-CD11b/c-PE (#554862, BD Pharmingen, USA), anti-CD34-PE (#sc-7324, Santa Cruz Biotechnology, USA), anti-CD29-FITC (#102205, Biolegend, USA), and anti-CD90-FITC (#561973, BD Pharmingen, USA) antibodies for 30 minutes. The cell suspension was then centrifuged at 1000 rpm for 5 minutes. Finally, the cell suspension was transferred into a new detection tube, followed by the detection of cell surface antigen using flow cytometry (BD Biosciences, San Jose, CA, USA).

### Cell Transfection and Stable Cell Line Construction

The lentivirus constructs for WTAP overexpression or knockdown were purchased from GenePharma (Shanghai, China). Briefly, BMMSCs were seeded into 6-well plates and infected with WTAP overexpression lentivirus, negative control, WTAP knockdown lentivirus, and scramble control at 50% confluence. The appropriate viral titer was determined according to the manufacturer’s instructions. After 72 h of transfection, stably transfected cell lines were selected with 2 μg/mL puromycin. The miRNA mimics and inhibitors were purchased from GenePharma (Shanghai, China) and were transfected into BMMSCs at a final concentration of 20 μM using Lipofectamine 2000 reagent (#11668030, Invitrogen, Carlsbad, CA, USA). All lentivirus constructs, mimics, negative control, and inhibitor sequences are listed in Supplementary Table S2.

### In Vivo Animal Experiments

WTAP-carrying lentivirus for overexpression (OE WTAP) and empty vector-carrying lentivirus (OE NC) were constructed by GenePharma (Shanghai, China). Knee arthrotomy and intramedullary injection of the femur were performed for virus delivery. Briefly, a 5 mm-longitudinal incision was made along the medial aspect of the quadriceps--patellar complex. The patella was dislocated laterally to expose the intercondylar groove. A 0.9 mm k-wire on a trephine drill was used to create a trephination defect. Internal pressure syringe was inserted through the defect, and 15 μL of WTAP lentivirus (5 × 10^7^ copies/mL) was injected into the intramedullary cavity. The quadriceps-patellar complex was then repositioned, and the medial arthrotomy was carefully repaired with 5-0 vicryl suture. Following the same surgical procedure as above, 15 L of an empty vector-carrying lentivirus (5 × 10^7^ copies/mL) was administered to the control group. The lentivirus as an in vivo delivery system can carry a luciferase transgene. The delivery system was injected into the marrow cavity of the femur 4 times at week 1, and luciferase expression in live mice was followed with a whole-animal fluorescence imaging system (IVIS Spectrum, USA) at 3 weeks post-injection.

### Microcomputed Tomography (Micro-CT) Analysis

The femurs were fixed with 4% paraformaldehyde at 4 °C for 2 days and then soaked in 75% ethanol at 4 °C before scanning and analysis. Images of the distal femurs were obtained by a Quantum GX microCT scanner (PerkinElmer, Inc., Waltham, MA). We analyzed the bone volume ratio (BS/BV), trabecular bone separation (Tb. Sp), bone mineral density (BMD), volume per tissue volume (BV/TV), trabecular bone number (Tb. N), and trabecular thickness (Tb. Th) values by Analyze 12.0 (PerkinElmer, Inc., Waltham, MA, USA).

### H&E and Masson Staining

The femurs of mice from the sham, OVX, OVX+OE NC, and OVX+OE WTAP groups were obtained 2 months after the treatment. The femoral specimens were soaked in 4% paraformaldehyde at 4 °C for 24 h and decalcified in 10% EDTA at room temperature for 21 days. Then the femurs were embedded in paraffin and sectioned (3-μm slices) along the longitudinal axis. The tissue sections were stained with H&E and Masson staining.

### Tartrate-Resistant Acid Phosphatase (TRAP) Staining

The femoral tissues were soaked in 4% paraformaldehyde for 24 h at 4 °C and decalcified in 10% EDTA for 3 weeks. Subsequently, the bone samples were dehydrated and embedded in paraffin. To quantify osteoclasts, TRAP staining was performed on sections from each group. Multinucleated TRAP-positive cells attached to femoral bone surfaces were imaged under a microscope (ZEISS, Axio Vert.A1, Germany).

### RNA Immunoprecipitation (RIP) Assay

RIP assays were performed using the Magna RIP RNA-Binding Protein Immunoprecipitation Kit (#17-704, Millipore, USA) according to the manufacturer’s instructions. Briefly, stably transfected BMMSCs were harvested and lysed in RIP lysis buffer. The anti-DGCR8 antibody (#ab191875, Abcam, USA), anti-m6A antibody (#ab286164, Abcam, USA), or anti-immunoglobulin G (IgG, #SAB5600195, Millipore, USA) was separately conjugated with magnetic beads. Then, the RIP lysate was incubated with magnetic beads at 4 °C overnight. The immunoprecipitated RNAs were extracted using TRIzol (#15596026, Invitrogen, Carlsbad, CA, USA), and the RNA enrichment was analyzed by qRT-PCR.

### Dual-Luciferase Reporter Assay

A dual-luciferase reporter assay was performed to verify that miR-29b-3p directly targets HDAC4 mRNA. The luciferase vector was constructed containing either the wild-type (WT) HDAC4 3ʹ untranslated region (UTR) or an HDAC4 3ʹUTR mutant (MUT) sequence. The BMMSCs were then cotransfected with the luciferase vector and miRNA mimics or its control (mimic-NC) using lipofectamine 2000 (#11668030, Invitrogen, Carlsbad, CA, USA). At 24 h after transfection, firefly and Renilla luciferase activities were analyzed by the Luciferase Reporter Assay System (Promega, Massachusetts, USA).

### Statistical Analysis

Data are presented as the mean ± SD, and SPSS 22.0 software was used for statistical analyses. Two independent groups were compared via Student’s *t*-tests, and comparisons between multiple groups were performed by one-way ANOVA. A *P* value of < .05 indicated statistical significance.

## Results

### WTAP Was Downregulated in Osteoporotic Bone Tissues and Was Correlated With the Differentiation of BMMSCs

**Figure 1.**
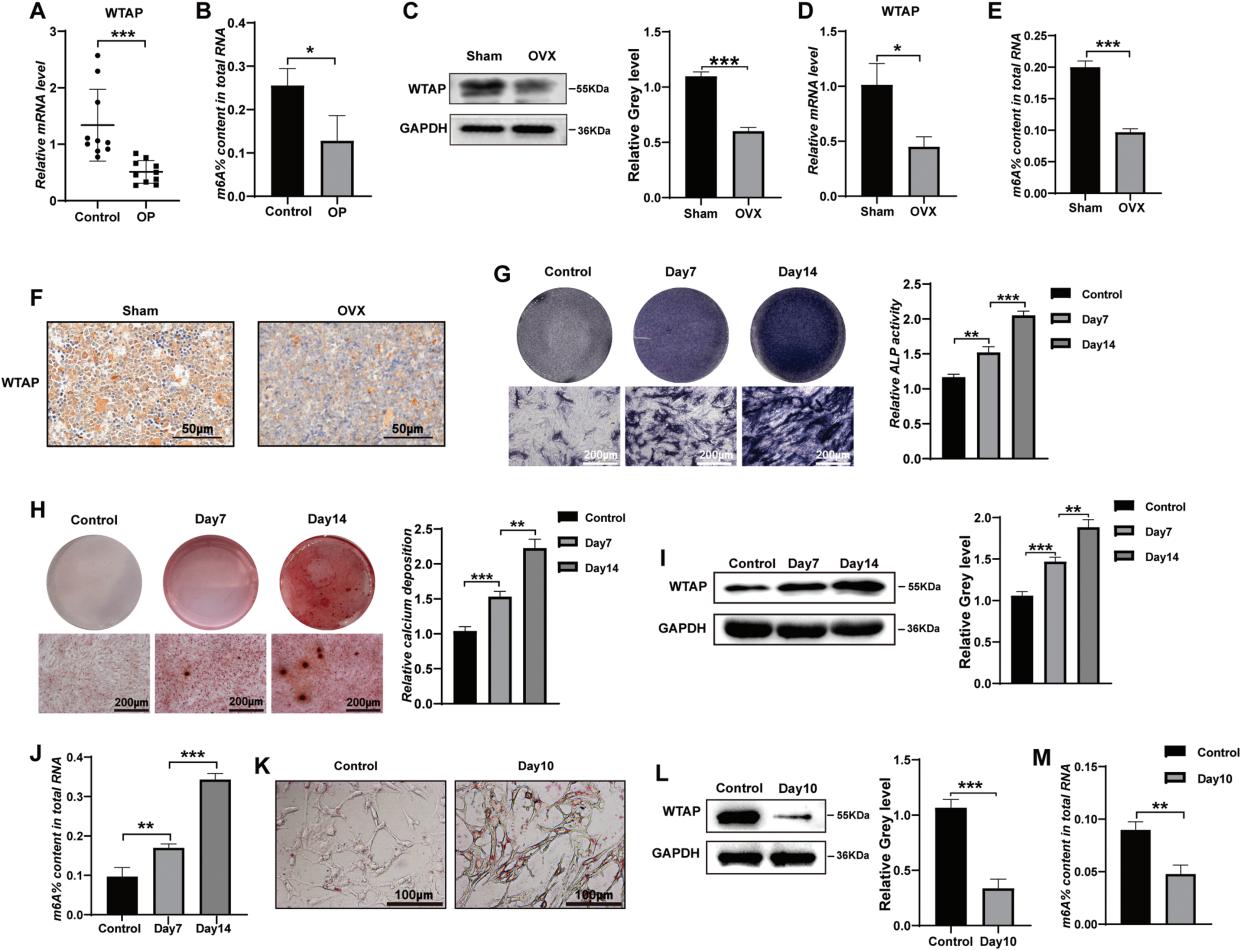
WTAP and m6A modification were decreased in osteoporotic bone tissues and were closely associated with the differentiation of BMMSCs. (**A**) qRT-PCR analysis of WTAP mRNA expression in 10 paired osteoporotic bone tissues and normal bone tissues. **(B)** The m6A level in total RNA was measured in bone tissues of the patients with OP. **(C, D)** The expression of WTAP in bone tissues from the OVX mice and the sham-operated control counterparts was determined using Western blot and qRT-PCR analyses. **(E)** The m6A level in total RNA was detected in the bone tissues of the OVX mice. **(F)** IHC analysis of WTAP protein expression in the femur marrow from the OVX mice and the sham control mice. **(G, H)** ALP staining and ARS were performed on days 7 and 14 during osteogenic differentiation. **(I, J)** WTAP expression at the protein level and the m6A level in total RNA were detected on days 7 and 14 during osteogenic differentiation. **(K)** Oil Red O staining was performed on day 10 of adipogenic differentiation. **(L, M)** WTAP protein expression and the m6A content in total RNA were measured on day 10 during adipogenic differentiation. Data are expressed as the mean ± SEM, *P < .05, **P < .01, ***P < .005.

We initially examined the expression patterns of the m6A methyltransferase WTAP in the bone tissue of patients with OP and OVX mice compared to control human subjects and sham-operated control mice, respectively. WTAP mRNA expression and the m6A content in total RNA were significantly decreased in the bone tissues of the OP patients (Fig. 1A, 1B). Similarly, the protein and mRNA levels of WTAP in the OVX mice also decreased significantly, compared with those in the sham groups (Fig. 1C, 1D). The m6A level in total RNA was decreased in bone tissues of the OVX mice (Fig. 1E). IHC analysis of bone samples indicated that the positive WTAP rate was significantly lower in the OVX mice (Fig. 1F). The BMMSCs had a fusiform shape and exhibited a vortex distribution (Supplementary Fig. S1A). Expression of the cell surface antigens CD11b/C, CD34, CD29, and CD90 was detected by flow cytometry. The results showed that the cells were negative for CD11b/C (<5%) and CD34 (<5%) and positive for CD29 (>95%) and CD90 (>95%) (Supplementary Fig. S1B). To confirm the osteogenic capacity of BMMSCs, we seeded the cells in 6-well plates for osteogenic induction. After induction for 14 days, ALP staining and ARS results showed more pronounced ALP activity and enhanced mineral deposition (Fig. 1G, 1H). Subsequently, we explored the expression levels of WTAP and m6A during osteogenic differentiation of BMMSCs. WTAP protein expression and the m6A content in total RNA were significantly increased during osteogenic differentiation (Fig. 1I, 1J). The results revealed that WTAP expression and m6A contents were positively correlated with the osteogenic ability of BMMSCs. Similarly, the cells were induced toward adipogenesis with adipogenic induction media. Oil Red O staining showed an increased number of oil red O-positive adipocytes at 10 days (Fig. 1K). WTAP protein expression and the m6A content in total RNA were significantly decreased during adipogenic differentiation (Fig. 1L, 1M). The results confirmed that WTAP expression and m6A contents were negatively correlated with the adipogenic ability of BMMSCs.

### Overexpression of WTAP Promoted Osteogenesis and Suppressed Adipogenesis of BMMSCs In Vitro

**Figure 2.**
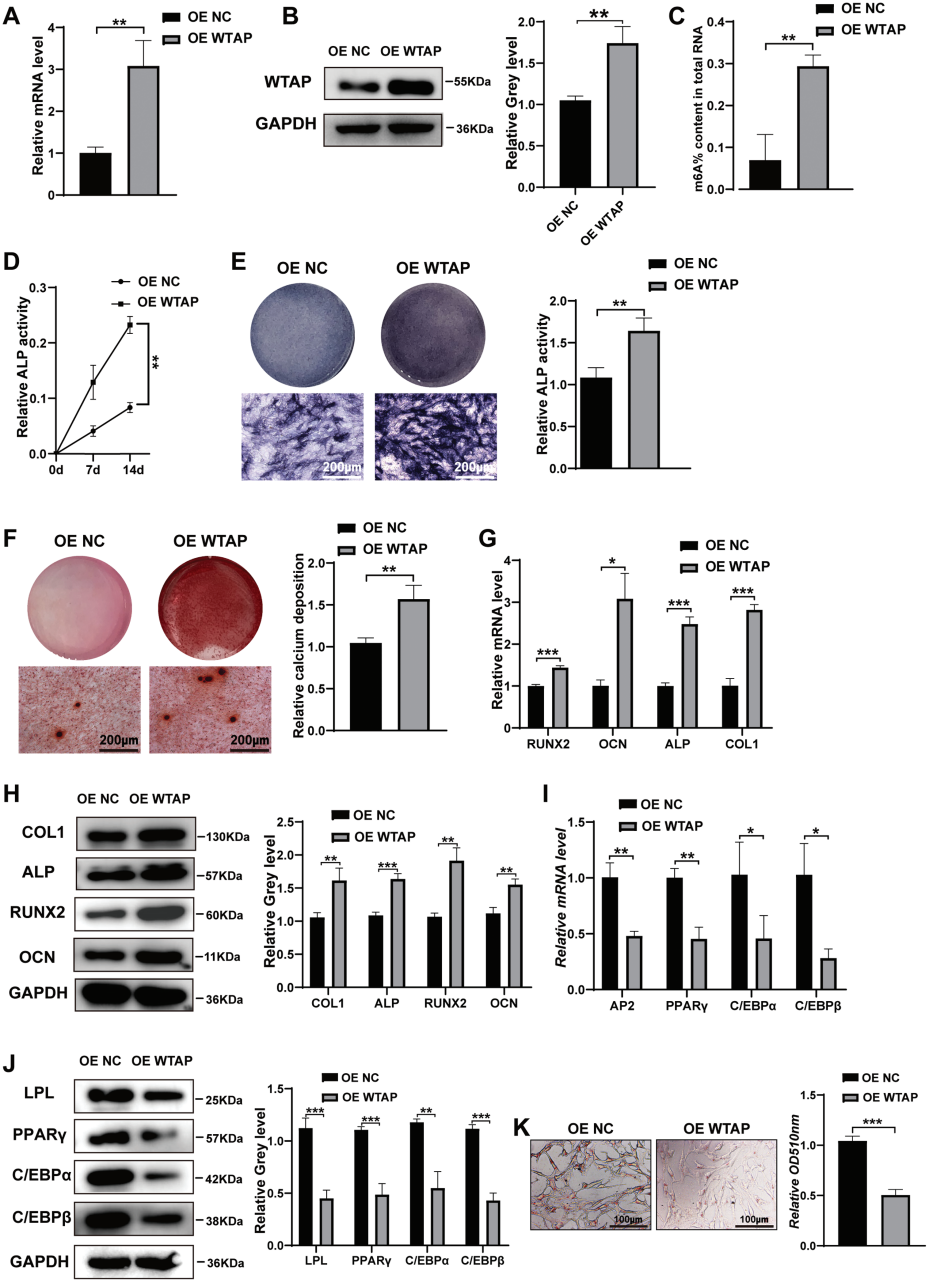
Overexpression of WTAP promoted osteogenic differentiation and inhibited adipogenic differentiation in vitro. (**A, B**) WTAP was measured by qRT-PCR and Western blot analysis after WTAP overexpression in BMMSCs. **(C)** The m6A content was measured in the WTAP-overexpressing BMMSCs. **(D)** ALP activity was detected after WTAP overexpression during osteogenic differentiation. **(E, F)** ALP staining and ARS were performed on day 14. **(G, H)** qRT-PCR and Western blot were performed to analyze the mRNA and protein levels of osteogenic-specific markers after WTAP overexpression. **(I, J)** The expression of adipogenic-specific markers was analyzed by qRT-PCR and Western blot. **(K)** Oil Red O staining and extraction were used to analyze the formation of lipid droplets on day 10 of adipogenic differentiation. Data are expressed as the mean ±SEM, *P < .05, **P < .01, ***P < .005.

WTAP expression was confirmed by qRT-PCR and Western blotting in stably transfected BMMSC lines (Fig. 2A, 2B). Owing to the m6A methylation function of WTAP, the relative m6A level increased after WTAP overexpression (Fig. 2C). The OE WTAP-infected BMMSCs were induced to differentiate into osteoblasts by osteogenic induction media. We found that ALP activity was enhanced in the OE WTAP-infected BMMSCs during osteogenic differentiation (Fig. 2D). ALP staining and ARS showed that the ALP activity and calcium nodule formation of BMMSCs were significantly increased after WTAP overexpression at 14 days (Fig. 2E, 2F). qRT-PCR and Western blot analysis showed that overexpression of WTAP resulted in higher levels of COL1, ALP, RUNX2, and OCN (Fig. 2G, 2H). However, the adipogenic differentiation of BMMSCs was significantly decreased after WTAP overexpression. The OE WTAP-infected BMMSCs showed decreased mRNA levels of AP2, PPARγ, C/EBPα, and C/EBPβ after WTAP overexpression (Fig. 2I). The protein levels of LPL, PPARγ, C/EBPα, and C/EBPβ were also significantly decreased (Fig. 2J). Oil Red O staining revealed that the number of lipid droplets was decreased in the OE WTAP groups (Fig. 2K).

### WTAP Knockdown Inhibited Osteogenic Differentiation and Facilitated Adipogenic Differentiation of BMMSCs In Vitro

BMMSCs were stably transfected with knockdown lentivirus and control lentivirus. The expression of WTAP was then confirmed using Western blotting and qRT-PCR (Supplementary Fig. S2A, S2B). The relative m6A contents decreased after WTAP knockdown (Supplementary Fig. S2C). Stably transfected BMMSCs were induced to differentiate into the osteogenic lineage. Depletion of WTAP significantly decreased ALP activity during osteogenic differentiation (Supplementary Fig. S2D). Knockdown of WTAP markedly decreased ALP staining and matrix mineralization (Supplementary Fig. S2E, S2F). Moreover, the qRT-PCR and Western blot results showed that knockdown of WTAP decreased osteogenic transcription factors and markers (Supplementary Fig. S2G, S2H). However, depletion of WTAP obviously increased adipogenesis-specific factors and marker genes at the mRNA and protein levels (Supplementary Fig. S2I, S2J). Oil Red O staining showed that WTAP knockdown promoted the formation of lipid droplets (Supplementary Fig. S2K).

### WTAP Overexpression Promoted Bone Formation in OVX Mice

**Figure 3.**
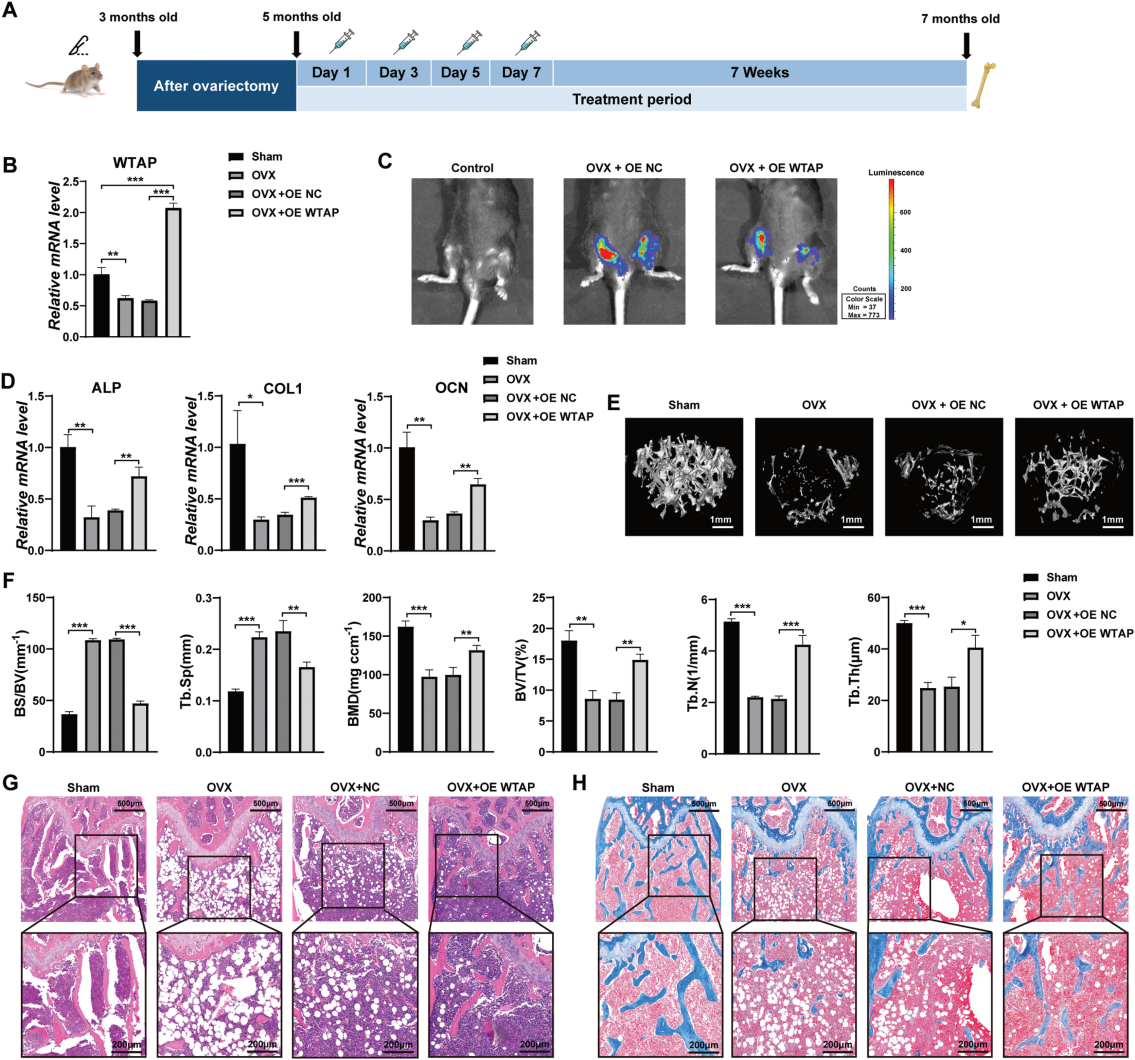
Overexpression of WTAP promoted bone formation in OVX mice. (**A**) Schematic diagram illustrating the experimental design. **(B)** Verification of the efficiency of WTAP overexpression at the mRNA level on day 7 after lentivirus injection in mice. **(C)** Live animal fluorescence imaging analysis of luciferase expression in the WTAP-overexpressing group and empty vector-carrying group. **(D)** qRT-PCR analysis of ALP, COL1, and OCN mRNA levels in bone specimens from mice at 7 weeks post-injection. **(E)** Representative images showing the 3-dimensional trabecular architecture in distal femurs determined by micro-CT reconstruction. **(F)** Micro-CT measurements of BS/BV, Tb. Sp, BMD, BV/TV, Tb. N, and Tb. Th in the distal femurs of mice after treatment with WTAP-overexpressing lentivirus or empty vector-carrying lentivirus. **(G)** H&E staining indicated trabecular density. **(H)** Masson trichrome staining indicated trabecular density and collagen. Data are expressed as the mean ± SEM, *P < .05, **P < .01, ***P < .005.

To determine whether WTAP is a direct regulator of m6A-mediated methylation in bone formation, we constructed a lentivirus delivery system for overexpressing WTAP through periosteal injection into the marrow cavity of the femur (Fig. 3A). We confirmed that WTAP mRNA expression was significantly increased in the mice bone tissue of mice at 7 days post-injection (Fig. 3B). To explore the effectiveness of the delivery system in mice, we tracked luciferase expression in the femurs of live mice at 3 weeks post-injection (Fig. 3C). In addition, BMMSCs were obtained from the femurs of mice and the WTAP expression of BMMSCs in the overexpressed WTAP group were significantly increased (Supplementary Fig. S3A, 3B). To further test the effects of WTAP on bone formation in vivo, we assessed the ALP, COL1, and OCN levels as indicators of osteoblast activity in mice at 7 weeks post-injection. The results showed that osteogenic transcription markers were markedly increased in bone tissue of WTAP-overexpressing mice (Fig. 3D). Micro-CT analysis revealed that the trabecular bone mass was significantly decreased (lower BMD and BV/TV), and the trabecular architecture was notably impaired (lower Tb. Th and Tb. N and higher Tb. Sp and BS/BV) in the OE NC-infected OVX mice, and these factors were partially rescued by WTAP overexpression (Fig. 3E, 3F). These alterations were also indicated by H&E and Masson’s trichrome staining (Fig. 3G, 3H). Together, these findings indicated that WTAP overexpression in OVX mice promoted bone formation.

### WTAP Regulated the Processing of miR-29b-3p

**Figure 4.**
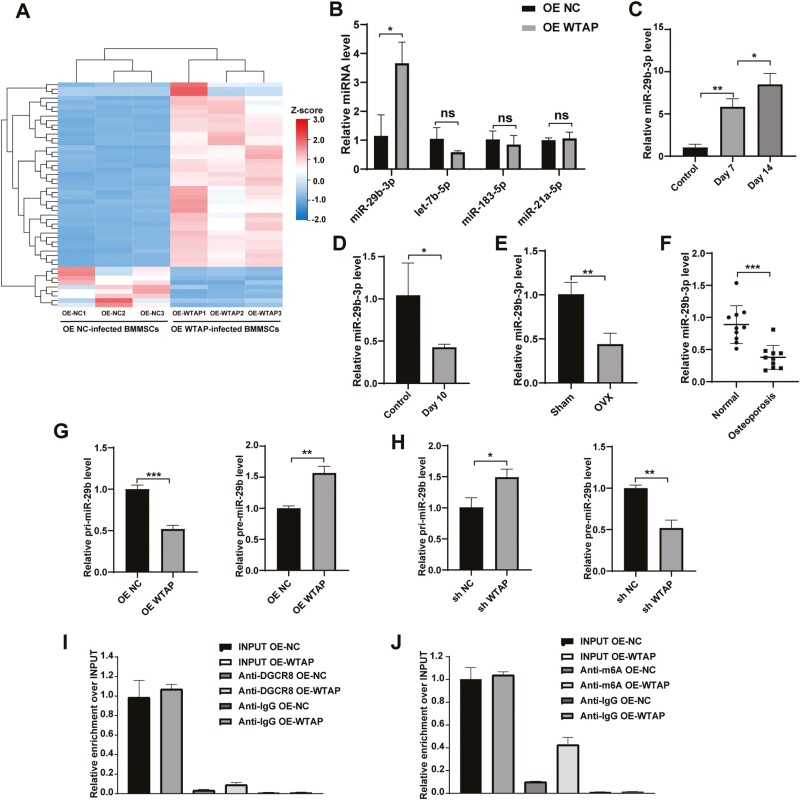
WTAP regulated the processing of miR-29b-3p. (**A**) The heatmap identified the differentially expressed miRNAs between the OE NC-infected BMMSCs and the WTAP-overexpressing BMMSCs (fold change > 2 or < −2, Benjamini-Hochberg-corrected *P*). **(B)** The expression of the differentially expressed miRNAs related to osteogenesis between the OE NC group and the OE WTAP group was measured by qRT-PCR. **(C)** qRT-PCR analysis of miR-29b-3p expression on days 7 and 14 during osteogenic differentiation. **(D)** The miR-29b-3p expression on day 10 of adipogenic differentiation was measured by qRT-PCR. **(E, F)** Decreased miR-29b-3p expression in bone tissues of the OVX mice and the patients with OP relative to that in the control counterparts. **(G, H)** The relative expression of primiR-29b and premiR-29b was measured by qRT-PCR. **(I)** qRT-PCR analysis of primiRNA binding to DGCR8 via RNA immunoprecipitation from the WTAP-overexpressing BMMSCs and the negative control BMMSCs. (**J**) qRT-PCR analysis of primiRNA modified by m6A via m6A immunoprecipitation from the WTAP-overexpressing BMMSCs and the negative control BMMSCs. Data are expressed as the mean ± SEM, *P < .05, **P < .01, ***P < .005.

To identify miRNAs differentially expressed between the OE NC-infected BMMSCs and the WTAP-overexpressing BMMSCs, we performed a miRNA microarray analysis to compare the miRNA expression profiles therein. The microarray results revealed significantly distinct miRNA profiles between the OE NC-infected BMMSCs and the OE WTAP-infected BMMSCs (Fig. 4A). Among the genes, miR-29b-3p, let-7b-5p, miR-183-5p, and miR-21a-5p were related to osteogenesis. Subsequently, the expression of these osteoblast-related genes was validated via qRT-PCR in the OE NC-infected BMMSCs and the OE WTAP-infected BMMSCs, revealing that miR-29b-3p was significantly upregulated in the OE WTAP groups relative to the OE NC groups (Fig. 4B).

qRT-PCR analysis showed that miR-29b-3p expression was strongly increased during osteogenic differentiation and decreased during BMMSC adipogenic differentiation (Fig. 4C, 4D). Moreover, the miR-29b-3p expression in the bone tissue of the OP patients and the OVX mice was significantly decreased compared to that in the corresponding control groups (Fig. 4E, 4F). Additionally, unprocessed primiR-29b was found to decrease in the WTAP-overexpressing cells and accumulate in the WTAP knockdown cells, whereas the opposite results were obtained for the premiR-29b level (Fig. 4G, 4H).

DGCR8 was immunoprecipitated from the WTAP-overexpressing BMMSCs and the negative control BMMSCs. The expression of DGCR8-bound primiR-29b was significantly increased in the WTAP-overexpressing BMMSCs (Fig. 4I). Additionally, m6A was immunoprecipitated from the WTAP-overexpressing cells and the negative control cells, revealing that WTAP overexpression significantly increased the expression of primiR-29b modified by m6A (Fig. 4J). These results showed that WTAP regulates the processing of miR-29b-3p in an m6A-dependent manner.

### miR-29b-3p Regulated BMMSC Differentiation

To further explore the biological function of miR-29b-3p in the osteogenic and adipogenic differentiation of BMMSCs, we used miR-29b-3p mimics and inhibitors to change the expression of miR-29b-3p in BMMSCs. The qRT-PCR results showed that miR-29b-3p expression was upregulated after transfection with miR-29b-3p mimics and significantly decreased after transfection with miR-29b-3p inhibitors (Supplementary Fig. S4A). Then, the BMMSCs transfected with the miR-29b-3p mimics and inhibitors were induced into the osteoblast lineage in osteogenic induction medium. Western blot and qRT-PCR analyses indicated that the expression of COL1, ALP, RUNX2, and OCN was significantly increased by miR-29b-3p mimic infection, while these osteogenic transcription factors and markers were downregulated after transfection with miR-29b-3p inhibitors (Supplementary Fig. S4B, S4C). In addition, ALP activity was increased in the miR-29b-3p-mimic-infected BMMSCs during osteogenic differentiation (Supplementary Fig. S4D). Overexpression of miR-29b-3p markedly increased ALP activity and calcium nodules, while inhibition of miR-29b-3p significantly decreased these osteogenic capabilities (Supplementary Fig. S4E, S4F). The BMMSCs transfected with the miR-29b-3p mimics and inhibitors were induced toward the adipogenic lineage with specific induction medium. The expression of adipogenic factors was decreased at the mRNA and protein levels by upregulation of miR-29b-3p and increased by miR-29b-3p knockdown (Supplementary Fig. S4G, S4H). Oil Red O staining and extraction indicated that overexpression of miR-29b-3p resulted in a significant decrease in the Oil Red O-positive adipocyte number, whereas inhibition of miR-29b-3p increased these adipogenic characteristics (Supplementary Fig. S4I).

### Downregulation of miR-29b-3p Reversed the Differentiation Induced by WTAP Overexpression

**Figure 5.**
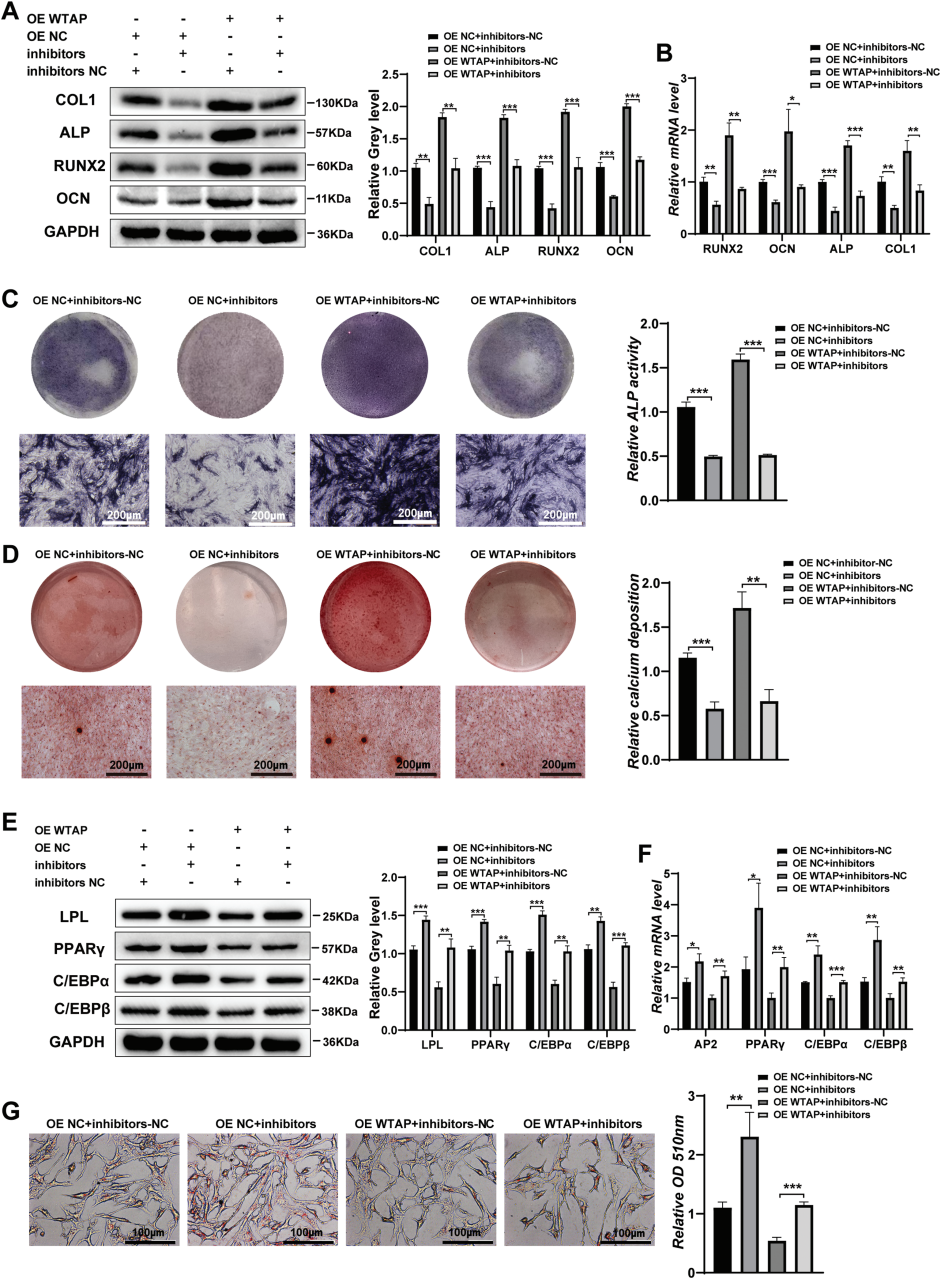
miR-29b-3p knockdown reversed the differentiation induced by WTAP overexpression. (**A**, **B**) The effect of miR-29b-3p inhibitors on osteogenic differentiation was measured by Western blot and qRT-PCR after WTAP overexpression. **(C, D)** The effect of miR-29b-3p inhibitors on osteogenic differentiation was measured by ALP staining and ARS after WTAP overexpression. **(E, F)** The effect of miR-29b-3p inhibitors on adipogenic differentiation was measured by Western blot and qRT-PCR after WTAP overexpression. **(G)** The formation of lipid droplets was analyzed by Oil Red O staining and extraction on day 10 of adipogenic differentiation. Data are expressed as the mean ± SEM, *P < .05, **P < .01, ***P < .005.

To further determine the effect of miR-29b-3p on the osteogenic and adipogenic differentiation of BMMSCs, we conducted a series of restoration assays. When miR-29b-3p inhibitors were transfected into the WTAP-overexpressing cells, we found that miR-29b-3p inhibitors could partly decrease the osteogenic differentiation increased by WTAP overexpression. The protein and mRNA levels of COL1, ALP, RUNX2, and OCN increased by WTAP were decreased by miR-29b-3p knockdown (Fig. 5A, 5B). Moreover, the increase in ALP staining and ARS induced by WTAP overexpression was reversed by miR-29b-3p knockdown (Fig. 5C, 5D). Our results also showed that the inhibition of miR-29b-3P reversed the effects of WTAP overexpression on adipogenic differentiation (Fig. 5E-5G).

### miR-29b-3p Rescued the Differentiation Induced by WTAP Knockdown

**Figure 6.**
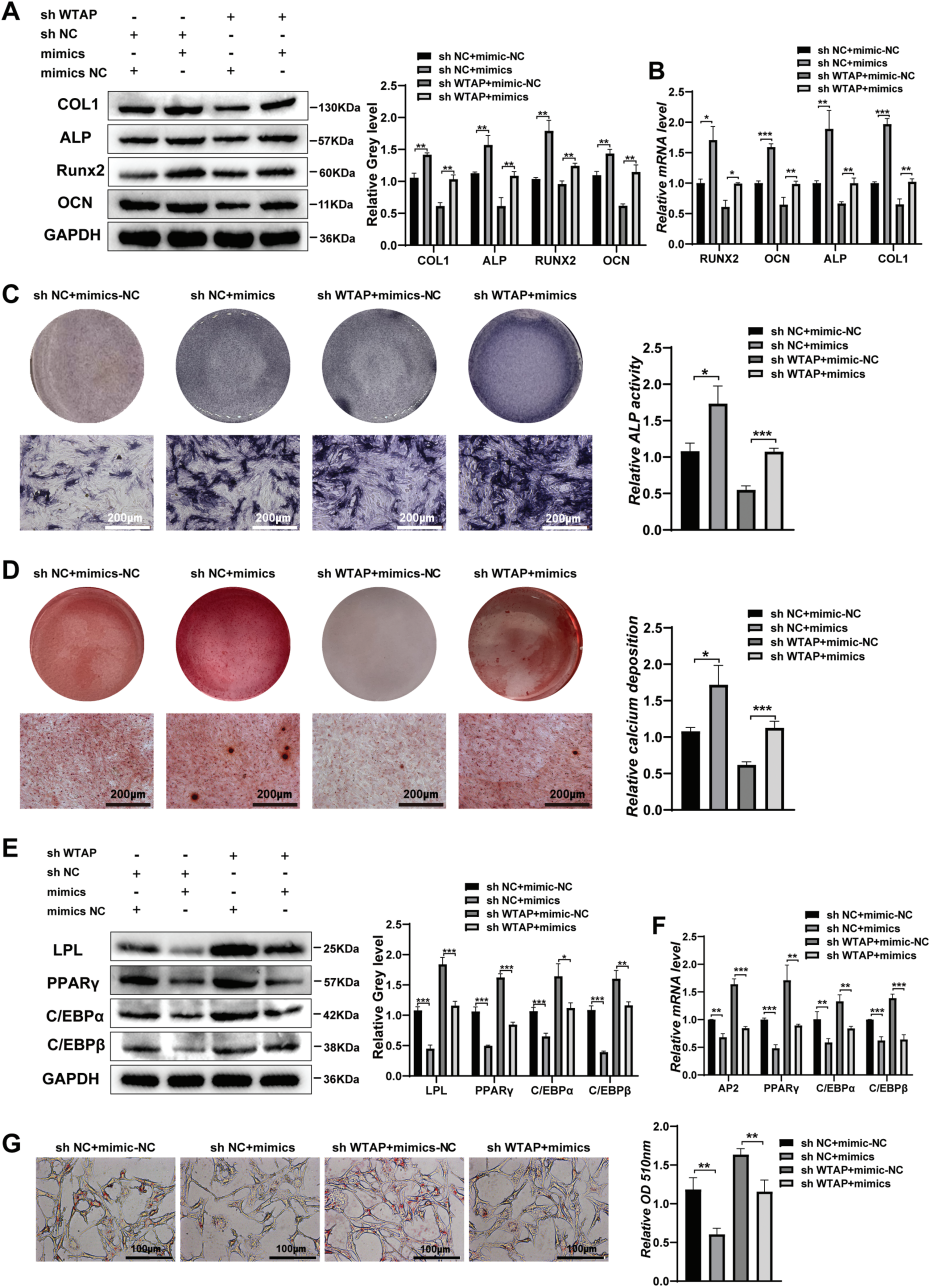
miR-29b-3p rescued the differentiation function induced by WTAP knockdown. (**A**, **B**) The effect of miR-29b-3p mimics on osteogenic differentiation was detected by Western blot and qRT-PCR after WTAP knockdown. (**C**, **D**) The effect of miR-29b-3p mimics on osteogenic differentiation was detected by ALP staining and ARS after WTAP knockdown. **(E, F)** The effect of miR-29b-3p mimics on adipogenic differentiation was detected by Western blot and qRT-PCR after WTAP knockdown. **(G)** Oil Red O staining and extraction were performed to analyze the formation of lipid droplets on day 10 of adipogenic differentiation. Data are expressed as the mean ± SEM, *P < .05, **P < .01, ***P < .005.

We upregulated miR-29b-3P in stable WTAP knockdown cells. The protein and mRNA levels of osteogenic transcription factors and markers decreased by WTAP knockdown were rescued by miR-29b-3P upregulation (Fig. 6A, 6B). Moreover, calcium nodule formation and ALP staining were increased by miR-29b-3p overexpression (Fig. 6C, 6D). In addition, miR-29b-3p overexpression partly inhibited the increased adipogenic abilities induced by WTAP knockdown (Fig. 6E-6G).

### miR-29b-3p Targeted HDAC4 During Osteogenic and Adipogenic Differentiation of BMMSCs

**Figure 7.**
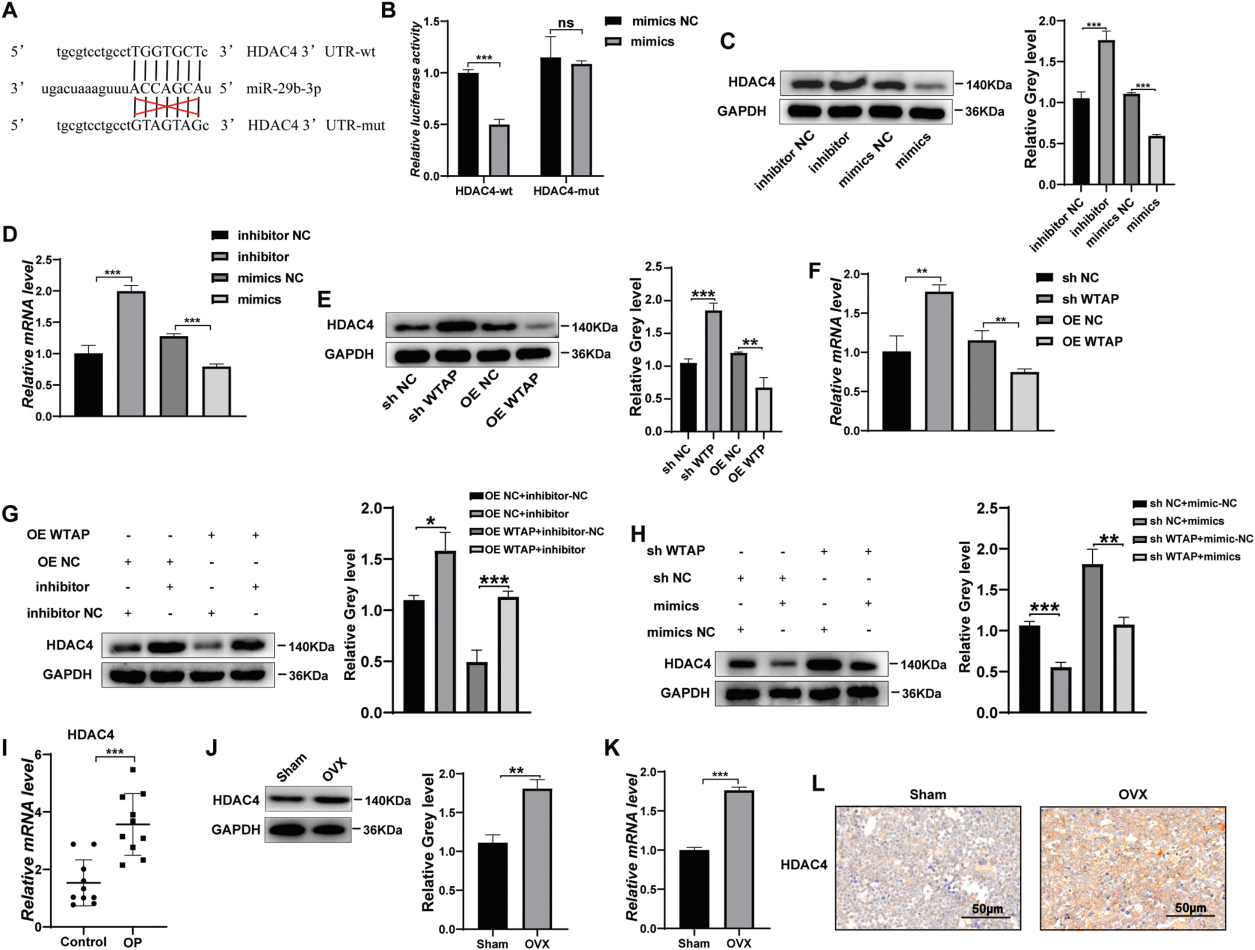
WTAP targeted HDAC4 via the WTAP/miR-29b-3p/HDAC4 pathways in BMMSCs differentiation. (**A**) miR-29b-3p and its predicted binding sequence in the wild-type (wt) and mutant (mut) 3ʹUTRs of HDAC4 mRNA. **(B)** Luciferase assays validated that HDAC4 is a target of miR-29b-3p. **(C, D)** The effect of miR-29b-3p mimics or inhibitors on HDAC4 expression was measured by Western blot and qRT-PCR. **(E, F)** HDAC4 expression was measured by Western blot and qRT-PCR in BMMSCs after WTAP knockdown or overexpression. **(G, H)** Western blot analysis showed that miR-29b-3p could partially rescue the expression of HDAC4 induced by WTAP. **(I)** HDAC4 mRNA expression in 10 paired osteoporotic bone tissues and normal tissues was determined by qRT-PCR. **(J, K)** The expression of HDAC4 in bone tissues from the OVX mice and the sham control mice was determined using Western blot and qRT-PCR. **(L)** IHC analysis of HDAC4 protein expression in the femur marrow from the OVX mice and the sham control mice. Data are expressed as the mean ± SEM, *P < .05, **P < .01, ***P < .005.

TargetScan (http://www.targetscan.org), miRDB (http://mirdb.org/), and ENCORI (https://starbase.sysu.edu.cn/) were used to predict the potential target genes of miR-29b-3P (Supplementary Fig. S5), and then we discovered that there were binding sites for miR-29b-3p in the 3ʹUTR of HDAC4 mRNA (Fig. 7A). Then, a dual-luciferase reporter assay was performed to verify whether HDAC4 is a direct target of miR-29b-3p. We found that miR-29b-3p overexpression significantly decreased the luciferase activity in the wild-type 3ʹUTR of HDAC4 compared with the mutant 3ʹUTR of HDAC4, suggesting that HDAC4 is a direct target of miR-29b-3p (Fig. 7B). In addition, we confirmed that HDAC4 expression was increased by miR-29b-3p knockdown and decreased by miR-29b-3p overexpression in BMMSCs (Fig. 7C, 7D). Moreover, HDAC4 expression was increased in the WTAP knockdown cells but was significantly decreased in the WTAP overexpression cells (Fig. 7E, 7F). Finally, the WTAP-overexpressing cells showed increased HDAC4 expression after miR-29b-3p inhibitor transfection into BMMSCs, while the transfection of the miR-29b-3p mimics partially reversed the upregulation of HDAC4 expression induced by WTAP downregulation (Fig. 7G, 7H). Furthermore, HDAC4 expression in bone tissues of both the OP patients and the OVX mice was higher than that in bone tissues from their control groups (Fig. 7I–7K). The IHC analysis of bone samples indicated that the positive rate of HDAC4 increased significantly in the OVX mice and decreased significantly in the sham control mice (Fig. 7L).

In summary, our research verified that WTAP regulates the differentiation of BMMSCs via the WTAP/miR-29b-3p/HDAC4 axis in an m6A-dependent manner.

### WTAP-Mediated m6A Methylation Negatively Regulates Osteoclast Differentiation

To explore the role of WTAP in the treatment of osteoporosis, we further explored the role of WTAP-mediated m6A methylation in bone resorption and osteoclasts. In vivo, TRAP-specific staining was performed to detect the osteoclast numbers of the mice femur. Compared to the OE-NC group, osteoclasts in the distal femur of WTAP overexpression mice were significantly decreased, suggesting that WTAP inhibited osteoclast differentiation and bone resorption in vivo (Supplementary Fig. S6A, S6B). In addition, the expression levels of osteoblast differentiation-related genes, including MMP-9, NFATc1, CTSK, and C-FOS were upregulated after 7 days of RANKL induction in RAW264.7 cells (Supplementary Fig. S6C, S6D). Furthermore, the mRNA and protein expression levels of WTAP were significantly reduced during osteoblast differentiation of RAW264.7 cells (Supplementary Fig. S6E, S6F). Meanwhile, the abundance of m6A in RAW264.7 cells was also reduced after 7 days of RANKL induction (Supplementary Fig. S6G). Additionally, WTAP knockdown significantly increased the expression of MMP-9, NFATc1, CTSK, and C-FOS (Supplementary Fig. S6H, S6I). The expression of m6A was decreased by WTAP knockdown in RAW264.7 cells (Supplementary Fig. S6J). WTAP overexpression in RAW264.7 cells resulted in a significant decrease of MMP-9, NFATc1, CTSK, and C-FOS (Supplementary Fig. S6K, S6L). WTAP overexpression increased the expression of m6A in RAW264.7 cells (Supplementary Fig. S6M). Taken together, WTAP-mediated m6A methylation plays an inhibitory role in osteoclast differentiation.

## Discussion

OP leads to an increased risk of fracture, subsequently impacting morbidity and mortality and the quality of life in postmenopausal women and older men.^1,4^ The imbalance between osteogenic and adipogenic differentiation of BMMSCs leads to the occurrence and development of OP.^6^ The inhibition of adipogenic differentiation and promotion of osteogenic differentiation of BMMSCs have become important methods for treating OP.^28^ Accumulating evidence has shown that m6A modification affects OP.^13,14,29^ The methyltransferase METTL3 and METTL14 have been shown to affect the potential of osteogenic differentiation in BMMSCs through distinct mechanisms.^13,29^ As one of the vital m6A methyltransferases, WTAP plays an indispensable role in disease progression by modifying RNAs,^16,17,21,30^ whereas the role of WTAP in OP remains unclear. In the present study, we found that WTAP promoted osteogenesis and inhibited adipogenesis in BMMSCs via the WTAP/miR-29b-3p/HDAC4 pathway by regulating the maturation of primiR-29b in an m6A-dependent manner. The hyperactivity of osteoclasts leads to increased bone resorption, which is further decomposed and absorbed by acids and enzymes secreted by osteoclasts, contributing to overall bone mass downregulation and leading to osteoporosis. Our study reveals that WTAP-mediated m6A methylation negatively regulates osteoclast differentiation. To our knowledge, we are the first to show that elevated WTAP expression is accompanied by an improved prognosis of OP.

METTL3 and METTL14 were reported to mediate miRNA maturation by interacting with DGCR8 in an m6A-dependent manner.^31-33^ Our study also confirmed that WTAP promoted the maturation of primiR-29b by interacting with DGCR8 and subsequently upregulated the expression of premiR-29b and mature miR-29b-3p in BMMSCs.

miR-29b-3p is considered a critical positive regulator of the osteogenic differentiation of MCS.^34-36^ Consistent with previous research, we found that osteogenic differentiation of BMMSCs was enhanced due to overexpression of miR-29b-3p. In addition, we first found that miR-29b-3p knockdown contributes to the positive regulation of adipogenic differentiation of BMMSCs.

HDAC4, as a crucial member of the HDAC family, was shown to be closely related to cell development and differentiation.^37^ HDAC4 induces deacetylation by removing acetyl groups from histones and non-histones through its zinc-containing catalytic structural domain.^38^ Jeon et al reported that HDAC4 could affect bone formation by regulating the non-histone protein deacetylation.^39^ Moreover, HDAC4 was shown to function as a negative regulator in osteoblasts.^40,41^ In our study, we verified that HDAC4 was upregulated in bone specimens from female OP patients and OVX mice. The miRNA-HDAC4 axis, a good target for disease treatment, plays a crucial role in cell growth and pathology.^37^ In the present study, the luciferase reporter assay confirmed that miR-29b-3p could bind to the 3ʹUTR of HDAC4 mRNA in BMMSCs. Further functional studies also verified that miR-29b-3p promoted osteogenic differentiation and inhibited adipogenic differentiation by directly targeting HDAC4. We first revealed that WTAP-mediated m6A modification influences osteogenic and adipogenic differentiation of BMMSCs through the miR-29b-3p/HDAC4 axis.

## Conclusion

In summary, our results showed that WTAP promoted osteogenesis and inhibited adipogenesis of BMMSCs via the WTAP/miR-29b-3p/HDAC4 pathways in an m6A-dependent manner. WTAP played a critical role in the osteogenic and adipogenic differentiation of BMMSCs through miRNA-mediated gene regulation. Furthermore, WTAP-mediated m6A methylation negatively regulates osteoclast differentiation of macrophages and further inhibits bone resorption. Given the functional importance of WTAP in bone formation, WTAP could serve as a potential therapeutic target in OP.

## Supplementary Material

szad020_suppl_Supplementary_Figure_S1

szad020_suppl_Supplementary_Figure_S2

szad020_suppl_Supplementary_Figure_S3

szad020_suppl_Supplementary_Figure_S4

szad020_suppl_Supplementary_Figure_S5

szad020_suppl_Supplementary_Figure_S6

szad020_suppl_Supplementary_Table_S1

szad020_suppl_Supplementary_Table_S2

## Data Availability

All relevant data and materials are available from the authors upon reasonable request.
