# Supplementary material for: WTAP-Mediated m6A RNA Methylation Regulates the Differentiation of Bone Marrow Mesenchymal Stem Cells via the miR-29b-3p/HDAC4 Axis
Source: Stem Cells Transl Med. 2023 Apr 3;12(5):307–21. doi: 10.1093/stcltm/szad020 (PMC10184703; doi:10.1093/stcltm/szad020)

Supplementary Figure S1. (A) Fusiform morphology of BMMSCs shown in light microscopy images. **(B)** Surface markers of BMMSCs analysed by flow cytometry. The cells were negative for CD34 and CD11b/C and positive for CD90 and CD29.

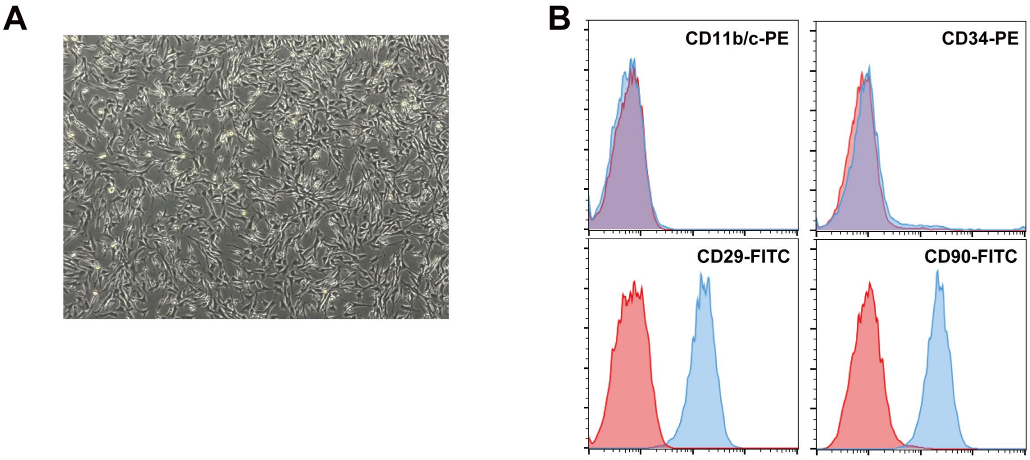

Supplement: szad020_suppl_Supplementary_Figure_S1 [file szad020_suppl_supplementary_figure_s1.pdf]
