# Supplementary material for: WTAP-Mediated m6A RNA Methylation Regulates the Differentiation of Bone Marrow Mesenchymal Stem Cells via the miR-29b-3p/HDAC4 Axis
Source: Stem Cells Transl Med. 2023 Apr 3;12(5):307–21. doi: 10.1093/stcltm/szad020 (PMC10184703; doi:10.1093/stcltm/szad020)
Supplement: szad020_suppl_Supplementary_Figure_S2 [file szad020_suppl_supplementary_figure_s2.pdf]

Supplementary data

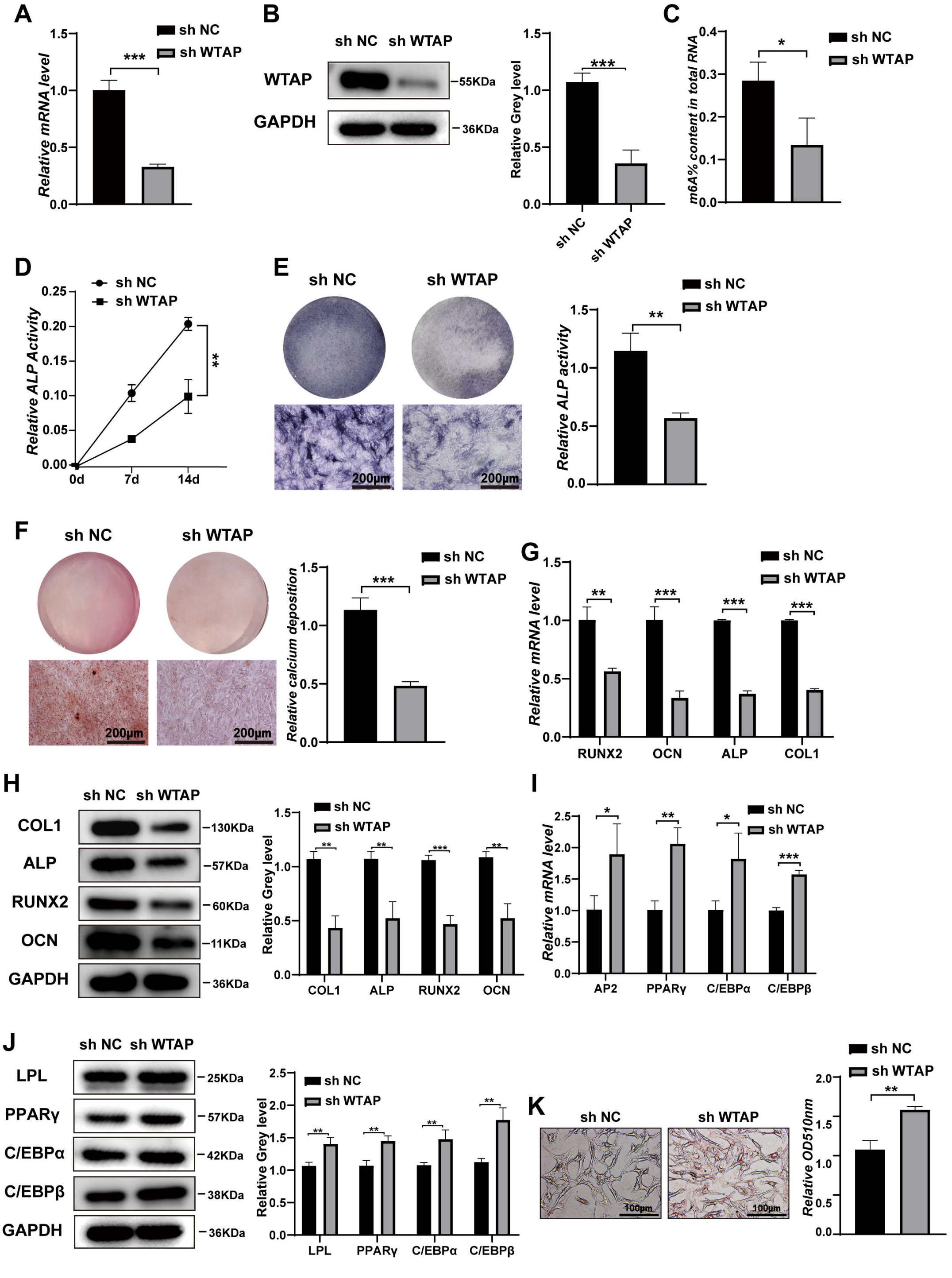

Supplementary Figure S2. Downregulation of WTAP suppressed osteogenic differentiation and enhanced adipogenic differentiation in vitro. **(A, B)** WTAP was measured by qRT-PCR and western blot analysis after WTAP knockdown in BMMSCs. **(C)** The m6A levels were measured in the WTAP knockdown BMMSCs. **(D)** ALP activity was detected after WTAP knockdown during osteogenic

differentiation. **(E, F)** ALP staining and ARS were performed on day 14. **(G, H)** qRT-PCR and western blot were performed to analyse the mRNA and protein levels of osteogenic-specific markers after WTAP knockdown. **(I, J)** WTAP knockdown increased the mRNA and protein expression levels of adipogenic-specific markers. **(K)** Oil red O staining and extraction were performed to detect the formation of lipid droplets on day 10 of adipogenic differentiation. Data are expressed as the mean ±SEM, *p <0.05, **p <0.01, ***p <0.005.
