# Supplementary material for: WTAP-Mediated m6A RNA Methylation Regulates the Differentiation of Bone Marrow Mesenchymal Stem Cells via the miR-29b-3p/HDAC4 Axis
Source: Stem Cells Transl Med. 2023 Apr 3;12(5):307–21. doi: 10.1093/stcltm/szad020 (PMC10184703; doi:10.1093/stcltm/szad020)
Supplement: szad020_suppl_Supplementary_Figure_S4 [file szad020_suppl_supplementary_figure_s4.pdf]

Supplementary data

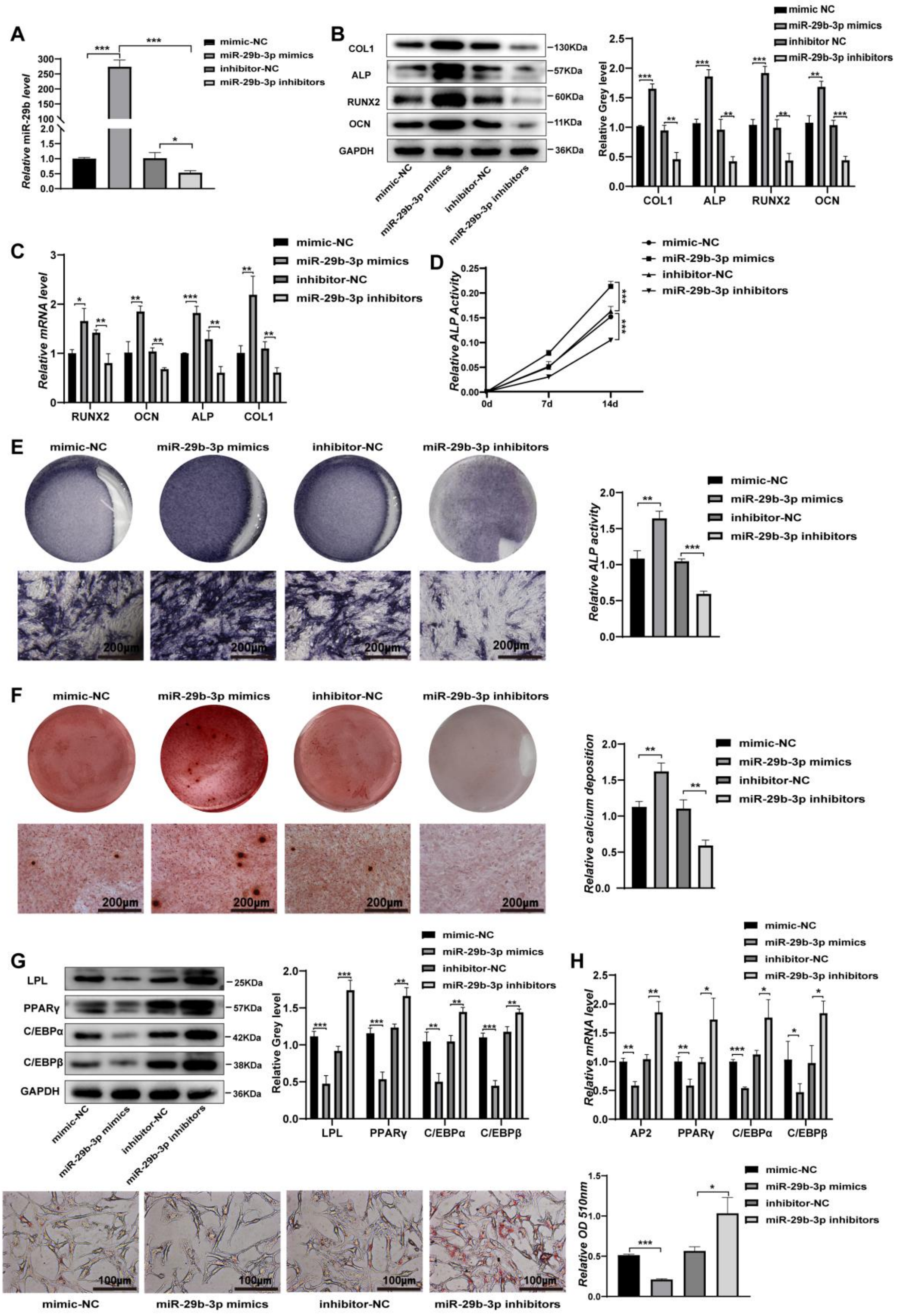

Supplementary Figure S4. MiR-29b-3p regulated BMMSC differentiation. (A) qRT-PCR analysis of miR-29b-3p levels after treatment with mimic-NC, miR-29b-3p mimics, inhibitor-NC, or miR-29b-3p inhibitors for 48 h. **(B, C)** Western blot and qRT-PCR were performed to analyse the protein and mRNA levels of osteogenic-specific markers after transfection. **(D)** ALP activity was detected after

transfection during osteogenic differentiation. **(E, F)** ALP staining and ARS were detected on day 14. **(G, H)** The expression of adipogenic-specific markers at the protein and mRNA levels was analysed by western blot and qRT-PCR. **(I)** Oil red O staining and extraction were used to analyse the formation of lipid droplets on day 10 of adipogenic differentiation after transfection. Data are expressed as the mean ± SEM, *$p < 0.05$, **$p < 0.01$, ***$p < 0.005$.
