# Supplementary material for: WTAP-Mediated m6A RNA Methylation Regulates the Differentiation of Bone Marrow Mesenchymal Stem Cells via the miR-29b-3p/HDAC4 Axis
Source: Stem Cells Transl Med. 2023 Apr 3;12(5):307–21. doi: 10.1093/stcltm/szad020 (PMC10184703; doi:10.1093/stcltm/szad020)
Supplement: szad020_suppl_Supplementary_Figure_S5 [file szad020_suppl_supplementary_figure_s5.pdf]

Supplementary Data

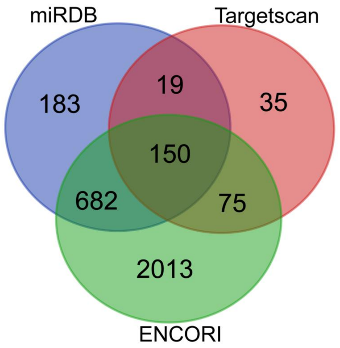

Supplementary Figure S5. Predicted target mRNAs arising from intersection outcome of miRDB, Targetscan and ENCORI.
