# Supplementary material for: WTAP-Mediated m6A RNA Methylation Regulates the Differentiation of Bone Marrow Mesenchymal Stem Cells via the miR-29b-3p/HDAC4 Axis
Source: Stem Cells Transl Med. 2023 Apr 3;12(5):307–21. doi: 10.1093/stcltm/szad020 (PMC10184703; doi:10.1093/stcltm/szad020)
Supplement: szad020_suppl_Supplementary_Figure_S6 [file szad020_suppl_supplementary_figure_s6.pdf]

Supplementary Data

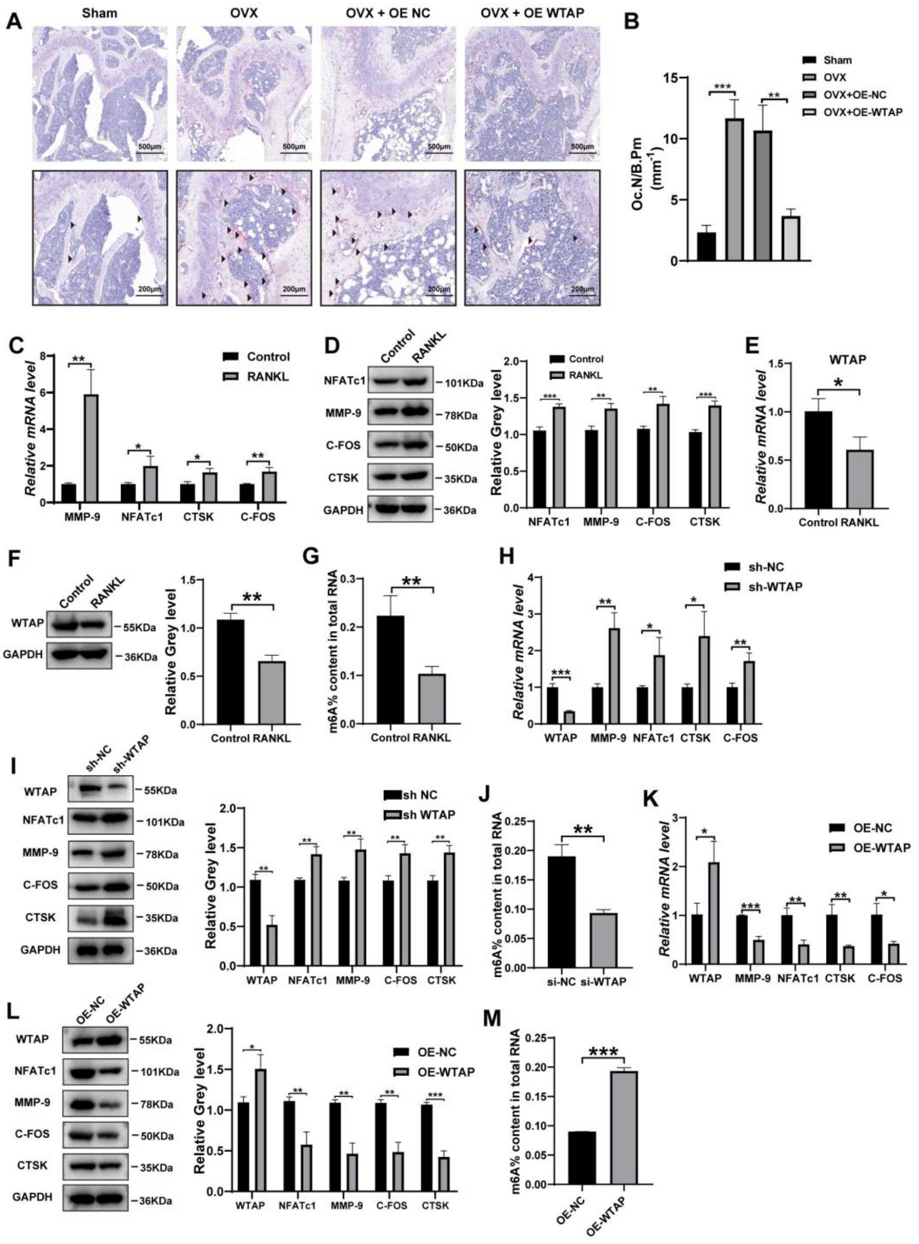

Supplementary Figure S6. WTAP-mediated m6A methylation negatively regulates osteoclast differentiation. (A, B) Representative images show tartrate-resistant acid phosphatase (TRAP) staining and osteoclast number per bone perimeter (Oc.N/B.Pm) in the femur sections of different groups. **(C, D)** Western blotting and qRT-PCR showed the upregulation of osteoclast marker genes (MMP-9, NFATc1, CTSK, C-FOS) with RANKL induced in RAW264.7 cells. **(E, F)** qRT−PCR and Western blotting were performed to analyse the mRNA and protein expression levels of WTAP during the osteoclast differentiation of RAW264.7 cells. **(G)** The m6A content was downregulated during the osteoclast differentiation of RAW264.7 cells. **(H, I)** qRT-PCR and Western blotting showed that WTAP knockdown increased the mRNA and protein expression levels of osteoclast marker genes (MMP-9, NFATc1, CTSK, C-FOS). **(J)** WTAP knockdown decreased the m6A content in

RAW264.7 cells. **(K, L)** qRT-PCR and Western blotting showed that WTAP overexpression inhibited the mRNA and protein expression levels of osteoclast marker genes (MMP-9, NFATc1, CTSK, C-FOS). **(M)** WTAP overexpression increased the m6A content in RAW264.7 cells.
