# Supplementary material for: WTAP-Mediated m6A RNA Methylation Regulates the Differentiation of Bone Marrow Mesenchymal Stem Cells via the miR-29b-3p/HDAC4 Axis
Source: Stem Cells Transl Med. 2023 Apr 3;12(5):307–21. doi: 10.1093/stcltm/szad020 (PMC10184703; doi:10.1093/stcltm/szad020)
Supplement: szad020_suppl_Supplementary_Table_S1 [file szad020_suppl_supplementary_table_s1.docx]

**Supplementary Table S1.** Primes sequences for qRT-PCR used in this study.

| Name | Sequence |
| --- | --- |
| *WTAP* Primer | F: AGTGCACCACTCAAATCCAGT  R: AGGCGTAAACTTCCAGGCA |
| *ALP* Primer | F: GCACCTGCCTTACCAACTCT  R: GTGGAGACGCCCATACCATC |
| *OCN* Primer | F: TCTGACCTCACAGATGCCAAG  R: AGGGTTAAGCTCACACTGCT |
| *OPN* Primer | F: CACATGAAGAGCGGTGAGTCT  R: CCCTTTCCGTTGTTGTCCTG |
| *RUNX2* Primer | F: GGGACTGTGGTTACCGTCAT  R: ATAACAGCGGAGGCATTTCG |
| *COL1* Primer | F: CCCTGGTCCCTCTGGAAATG  R: GGACCTTTGCCCCCTTCTTT |
| *GAPDH* Primer | F: GGTCACCAGGGCTGCTTTTA  R: GGATCTCGCTCCTGGAAGATG |
| *LPL* Primer | F: ACAAGAGAGAACCAGACTCCAA  R: AGGGTAGTTAAACTCCTCCTCC |
| *AP2* Primer | F: AGCACCATAACCTTAGATGGGG  R: CGTGGAAGTGACGCCTTTCA |
| *PPARγ* Primer | F: GCCGAGTCTGTGGGGATAAA  R: TCCGGCAGTTAAGATCACACC |
| *C/EBPα* Primer | F: AGGAACACGAAGCACGATCAG  R: CGCACATTCACATTGCACAA |
| *C/EBPβ* Primer | F: CTTCAGCCCGTACCTGGAG  R: GGAGAGGAAGTCGTGGTGC |
| *MMP-9* Primer | R:AGAAGAAAACCCTCTTGGTC  F:TGTTTTTGATGCTATTGCTG |
| *NFATc1* Primer | R:GGAGAGTCCGAGAATCGAGAT  F:TTGCAGCTAGGAAGTACGTCT |
| *CTSK* Primer | R:CTCGGCGTTTAATTTGGGAGA  F:TCGAGAGGGAGGTATTCTGAGT |
| *C-FOS* Primer | R:GCGAGCAACTGAGAAGAC  F:TTGAAACCCGAGAACATC |
| *miR-29b-3p* Primer | F: ACAGCAATTAGCACCATTTGAA  R: TATGCTTCTTCTCGTCTCTGTGTC |
| *pri-miR-29b* Primer | F: TCACCTTCCCTCTCCGTAGGAA  R: TCTAAACCACCATATGAAACCAGC |
| *pre-miR-29b* Primer | F: AGGAAGCTGGTTTCATATGGTGR  R: GAACACTGATTTCAAATGGTGCTAG |
| *U6* Primer | F: CAGCACATATACTAAAATTGGAACG  R: ACGAATTTGCGTGTCATCC |
| F, forward; R, reverse; WTAP, Wilms tumor 1-associated protein; ALP, Alkaline Phosphatase; OCN, osteocalcin; OPN, osteopontin; Runx2, runt-related transcription factor 2; COL, collagen; LPL, lipoprotein lipase; AP2, namely FABP4, fatty acid binding protein 4; PPARγ, peroxisome proliferator-activated receptor gamma; C/EBPα, CCAAT/enhancer binding proteinα; C/EBPβ, CCAAT/enhancer binding protein β; MMP9, matrix metallopeptidase 9; NFATc1, Nuclear factor of activated T cells 1; CTSK, cathepsin K; C-Fos, c-Fosproto-oncogene; | |
