# Supplementary material for: WTAP-Mediated m6A RNA Methylation Regulates the Differentiation of Bone Marrow Mesenchymal Stem Cells via the miR-29b-3p/HDAC4 Axis
Source: Stem Cells Transl Med. 2023 Apr 3;12(5):307–21. doi: 10.1093/stcltm/szad020 (PMC10184703; doi:10.1093/stcltm/szad020)
Supplement: szad020_suppl_Supplementary_Table_S2 [file szad020_suppl_supplementary_table_s2.docx]

**Supplementary Table S2.** Lentivirus constructs, mimics, and inhibitors sequences used in this study.

| Name | Sequence |
| --- | --- |
| shWTAP | CGGGATGAGTTA ATTCTAA |
| miR-29b-3p mimics | sense: UAGCACCAUUUGAAAUCAGUGUU  antisense: CACUGAUUUCAAAUGGUGCUAUU |
| miR-29b-3p mimic NC | sense: UUCUCCGAACGUGUCACGUTT  antisense: ACGUGACACGUUCGGAGAATT |
| miR-29b-3p inhibitors | AACACUGAUUUCAAAUGGUGCUA |
| miR-29b-3p inhibitor NC | CAGUACUUUUGUGUAGUACAA |
|  | |
